# Status and epidemiological characteristics of high-risk human papillomavirus infection in multiple centers in Shenyang

**DOI:** 10.3389/fmicb.2022.985561

**Published:** 2022-09-15

**Authors:** Di Yang, Jing Zhang, Xiaoli Cui, Jian Ma, Chunyan Wang, Haozhe Piao

**Affiliations:** ^1^Department of Gynecology, Liaoning Cancer Hospital and Institute, Cancer Hospital of China Medical University, Shenyang, China; ^2^Department of Obstetrics and Gynecology, Shengjing Hospital of China Medical University, Shenyang, China; ^3^Department of Neurosurgery, Liaoning Cancer Hospital and Institute, Cancer Hospital of China Medical University, Shenyang, China

**Keywords:** screening, human papillomavirus, high risk type, Shenyang area, epidemiological characteristics

## Abstract

The different human papillomavirus (HPV) strains cause warts in various regions of the body. However, considering that the status and genotype distribution of HPV infection in women in Shenyang remain unknown, herein, we investigated the epidemiological characteristics of high-risk HPV (HR-HPV) infection in women in Shenyang, as well as the current state of HPV infection in Shenyang, to provide a theoretical basis for the prevention and treatment of cervical cancer. From December 2018 to December 2021, 6,432 urban and rural women from the Liaoning Cancer Hospital and the Sujiatun Women and Infants’ Hospital were assessed via the Thinprep cytology test (TCT) and HR-HPV detection. Of the 5,961 women enrolled, 739 were HPV positive (12.40%) and 562 were TCT positive (9.43%). Statistical analyses identified the following HPV risk factors: high school education or lower [OR = 1.426 (1.199–1.696), *p* < 0.001], age at first sexual encounter ≤ 19 years [OR = 1.496 (1.008–2.220), *p* = 0.046], and number of sexual partners > 1 [OR = 1.382 (1.081–1.768), *p* = 0.010], atypical squamous cells of undetermined significance (ASCUS) and above [OR = 10.788 (8.912–13.060), *p* < 0.001], non-condom-based contraception [OR = 1.437 (1.103–1.871), *p* = 0.007], nationalities other than Han [OR = 1.690 (1.187–2.406), *p* = 0.004], rural residence [OR = 1.210 (1.031–1.419), *p* = 0.020]. Compared to the HPV infection rate of women aged 56–65, that in women aged 35–45 [OR = 0.687 (0.549–0.860), *p* = 0.001] and 46–55 [OR = 0.740 (0.622–0.879), *p* = 0.001] decreased significantly. To conclude, risk factors of HPV infection among female patients include high school age and below, initial sexual encounter at age ≤ 19 years, number of sexual partners > 1, ASCUS and above, non-condom contraception, nationalities other than Han nationality and rural population. Collectively, this study provides insights for the improved prevention and treatment of cervical cancer.

## Introduction

In women, cervical cancer ranks fourth among all cancers in incidence (13.1%) and mortality (6.9%), only after breast cancer, colorectal cancer, and lung cancer (Arbyn et al., 2020). In 2020, approximately 604,127 new cases were diagnosed worldwide, while 341,831 people died of cervical cancer (Sung et al., 2021). A total of 85% of cases occur in developing countries, one third of which occur in China and India. In developing countries, cervical cancer ranks second in incidence rate among female tumors, and first in mortality (Bray et al., 2018). Moreover, an increasing trend in incidence among younger individuals has been noted, thus posing a serious risk to women’s health (Bruni et al., 2019). Indeed, within 2020, 109,741 new cervical cancer cases and 59,060 cervical cancer-related deaths were reported in China (Zheng et al., 2019; Goodman et al., 2020).

Human papillomavirus (HPV), a double-stranded circular DNA virus, is a papillomavirus belonging to the family Papillomaviridae. To date, more than 100 HPV genotypes have been characterized, forty of which are associated with human reproductive tract infections via uncontrolled induction of squamous epithelia proliferation within the mucosa (Ardekani et al., 2022). HPV represents a common sexually transmitted infection worldwide. Approximately 75% of sexually active people will experience HPV infection in their lifetime (Trottier and Franco, 2006). Meanwhile, the main risk factor for cervical cancer and its precancerous lesions is persistent infection with high-risk HPV (HR-HPV) (de Sanjosé et al., 2018). Approximately 90% of cervical cancers contain DNA sequences of specific HPV genotypes with most cases caused by HR-HPV infection transmitted through sexual intercourse. Therefore, primary prevention (HPV vaccination) and secondary prevention (screening and treatment of cervical precancerous lesions) can effectively prevent cervical cancer (Arbyn et al., 2020). Therefore, the prevention and treatment of HR-HPV infection is an important way of preventing female cervical cancer.

Significant differences have been reported in HPV infection rates and types among people in different regions (Duan et al., 2022). For example, the positivity rate, as well as the distribution and characteristics of the HPV types, differ in women of different ages and regions. In particular, the HPV infection rate in women in China is 15.5%, which is lower than that in African countries (20.9–23.4%) and higher than that in other Asian countries (5.5–7.5%), Europe (7.8–8.4%), and North America (12.4–13.5%). Moreover, within China, differences in the HPV infection rate and subtype distribution have been reported among different regions and ethnic groups (Choi et al., 2018). For example, the infection rate is 33.05% in Hebei, 28.43% in Guangzhou, 27.09% in Fujian, 24.31% in Guizhou, and 16.64% in Shanghai (Solomon et al., 2002).

From a clinical point of view, epidemiological studies investigating HPV infection among women in different regions have been conducted to help reduce the prevalence of infection among women. However, to date, no study has reported on the status and genotype distribution of HPV infection in women in Shenyang. Therefore, the current cross-sectional study examines the prevalence and high-risk factors of HPV infection within women in Shenyang. Moreover, the epidemiological characteristics of HR-HPV in women in this region are assessed, and a novel strategy for the prevention and treatment of cervical cancer is proposed.

## Materials and methods

### Ethics statement

This study was approved by the Ethics Committee of the Liaoning Cancer Hospital (ethics batch number: 20180106), and informed consent was obtained from all participants included in this study.

### Research participants

From April 2018 through December 2021, 6,432 women in Liaoning were screened for cervical cancer at the Liaoning Cancer Hospital and the Shenyang Sujiatun District Women’s and Infant Hospital. The women selected for this study had an average age of 51.56 ± 6.621 years and a median age of 51 years. Community population and hospital outpatient opportunistic screenings were employed to evaluate whether participants met the following inclusion criteria: (1) the screening participants were selected by the community (village committee) and did not represent a single occupational group; (2) women from urban and rural populations with high incidence rates and mortalities and good population compliance were selected as the target population; (3) only participants with a registered residence in their regions (living in the local area for more than 3 years) were screened; (4) women with a history of sexual life, aged between 35 and 65 years old (subject to the date of birth on the ID card); and (5) women with no serious organ dysfunction or mental disease, who voluntarily participate in and can accept the questionnaire survey. Exclusion criteria for screening objects included the following: (1) Women who have been diagnosed with a tumor; (2) women who are currently being treated for other serious internal and external diseases; (3) women who have had a hysterectomy; and (4) women who are pregnant and lactating.

### Methods

The Thinprep cytology test (TCT) was combined with HPV detection technology to screen cervical lesions. Each medical examiner took the stone cutting position. The vulva was fully disinfected and the cervix exposed with a vaginal dilator. Next, the cervix was wiped with a sterile cotton swab to remove excess cervical secretions. Finally, a disposable cervical cell sampling brush was rotated ten times within the scaly columnar junction of the cervix, removed, and stored in a cell preservation solution.

### Liquid-based cytology

A TCT (Thinprep cytologic test purchased from Hologic.lnc 20160621) test was used to obtain materials for production. The accompanying report was prepared based on The Bethesda System (TBS) for Reporting Cervical Cytology (2014). Abnormal results included negative for intraepithelial lesion or malignant tumor (NILM), atypical squamous cells of undetermined significance (ASCUS), atypical squamous cells cannot exclude high-grade squamous intraepithelial lesion (ASCH), low-grade squamous intraepithelial lesion (LSIL), high grade squamous intraepithelial lesion (HSIL), squamous cell carcinoma (SCC), and atypical glandular cells (AGC). A positive TCT diagnosis included patients with ASCUS, ASC-H, LSIL, HSIL, SCC, or AGC.

### Human papillomavirus detection technology

The E6/E7 mRNA detection kit (Hologic Gen-Probe, San Diego, CA 20183401863)—an FDA-approved assay for detecting HPV—was used to detect 14 HR-HPV-mRNA causing cervical cancers (16, 18, 31, 33, 35, 39, 45, 51, 52, 56, 58, 59, 66, and 68). The results for HPV type 16 and 18 detection can be made available to provide clinical guidance. The kit detects E6 and E7, rather than L1, mRNAs of the HR-HPV virus to avoid the missed diagnosis of high-level lesions and cancer. Moreover, the test avoids cross-reactivity with low-risk HPV types, has fewer false positives, and avoids unnecessary colposcopy and overdiagnosis.

### Standard referral colposcopy

Cervical exfoliative cells were examined using cervical liquid-based TCT. The diagnostic criteria are based on the TBS classification as outlined above (Kclurman et al., 2014). Colposcopies were also performed in patients that were HPV positive and/or assigned a TBS classification of ASCUS or above, or found to have clinically suspicious abnormalities. If the results of a multi-point tissue biopsy for cervical lesions were suspicious, the bite tissues were sent for pathological examinations. The pathological results were of the gold standard. Diagnostic criteria included normal or inflammatory reactions, cervical intraepithelial neoplasia (CIN), and cervical cancer. Recently, CIN has gained secondary classifications based on the severity of the disease and can be classified on a scale from one to three (CINI-CINIII) (Liang et al., 2016).

### Technical quality control

For quality control purposes, 20% of the positive cervical exfoliative cell smears and 5–10% of the negative smears were randomly selected. All selected smears were rechecked by experts. The qualified rate of smear results reached 80%. Quality control of colposcopy samples entailed a spot check for 10% of the normal reports and 20% of abnormal reports, as well as subsequent analysis by experts. The standard rate of the report results reached 90%. Quality control of histopathological examination entailed a spot check of 10% of the pathological sections, subsequently rechecked by experts. The coincidence rate of the diagnostic results reached 90%.

### Investigation of risk factors and awareness of high-risk-human papillomavirus

Analysis was performed on the basic population information, as well as the living habits, physiological indicators, psychological and emotional conditions, screening willingness, and other high-risk factors. The questionnaire posed question regarding demographic characteristics and risk factors that may be associated with HR-HPV infections. Additionally, participants answered questions meant to reflect their level of knowledge about, and overall attitudes toward, cervical cancer, HPV, and the correlation between the two. All questions were answered directly by each participant or by dictating answers to a study nurse.

### Statistical analysis

Statistical analysis of the relevant data was conducted using the statistical package for the social science (SPSS) version 19.0 software. The count data rate (%) was analyzed using the chi-square (χ^2^) test. The influencing factors were analyzed using univariate and multivariate logistic regression to evaluate the correlation between the relevant factors mentioned in the questionnaire and HPV infection.

## Results

### Demographic characteristics

#### Basic population information

Of the 6,432 participants, 262 were 35–40 years old (4.40%), 941 were 41–45 years old (15.79%), 1,581 were 46–50 years old (26.52%), 1,443 were 51–55 years old (24.21%), 1,115 were 56–60 years old (18.70%), and 619 were 61–65 years old (10.38%; Table 1).

**TABLE 1:** Basic information of the cervical cancer screening population.

Characteristics		N	n (%)
Age (years)	35–40	262	4.40
	41–45	941	15.79
	46–50	1,581	26.52
	51–55	1,443	24.21
	56–60	1,115	18.70
	61–65	619	10.38
Ethnicity	Han	5,058	84.85
	Others	179	3.00
Marital status	Unmarried	146	2.45
	Married	5,578	93.57
	Divorced	168	2.82
	Widowed	68	1.14
Profession	Head of party and enterprise unit	423	7.10
	Professional skilled worker	1,064	17.85
	Office and related personnel	466	7.82
	Social production and life service personnel	974	16.34
	Agriculture, forestry, animal husbandry, fishery production, and auxiliary personnel	158	2.65
	Production and related personnel	408	6.84
	Soldiers	4	0.07
	Others who are difficult to classify	1,984	33.28
	Others	479	8.04
Educational level	Junior high school and below	1,990	33.38
	Senior high school	2,009	33.70
	College degree or above	1,961	32.90
Total		5,961	100.00

#### Personal and family history

Menarche age occurred between 12 and 18 years old in 97.94% of the participants. Menopause had occurred in 52.86% of the participants, most of whom were over 50 years of age. Moreover, 83.14% of the participants had a history of breast-feeding, and 71.26% breast-fed for more than 6 months. The population with multiple sexual partners accounted for 8.92 and 3.00% of the participants were aged < 19 years old at the time of their first sexual encounter. A total of 71.25% had a history of abortion; those with lover’s redundant prepuce and sexual bleeding accounted for 5.54 and 6.46%, respectively. A total of 22.18 and 17.55% women had a history of gynecological diseases and family histories of tumors, respectively (Table 2).

**TABLE 2:** Personal and family history of the cervical cancer screening population.

Characteristics		N	n (%)
Age at menarche (years)	<12	68	1.14
	12–18	5,838	97.94
	>18	55	0.92
Menopausal	Yes	3,151	52.86
	No	2,810	47.14
Age at menopause	<50	966	16.21
	≥50	2,185	36.65
Breastfeeding history	Yes	4,956	83.14
	No	1,005	16.86
Breastfeeding time	≤6 Months	708	11.88
	>6 Months	4,248	71.26
Sexual partners	0	11	0.18
	1	5,364	89.98
	≥2	532	8.92
Age at first sexual activity	Never	2	0.03
	≤19	179	3.00
	20–30	5,656	94.88
	≥31	68	1.14
Pregnancy history	Yes	5,810	97.47
	No	75	1.26
History of miscarriage	Yes	4,247	71.25
	No	1,571	26.35
Sexual partner’s foreskin is too long	Yes	323	5.42
	No	5,567	93.39
Bleeding during intercourse	Yes	385	6.46
	No	5,499	92.25
Cervical cancer vaccine	Yes	31	0.52
	No	5,858	98.27
Abnormal vaginal discharge	Yes	679	11.39
	No	5,146	86.33
History of gynecological disease	Yes	1,322	22.18
Family history of cancer	Yes	1,046	17.55

#### Living habits

Current smokers accounted for 8.37% of all participants, while those who had quit smoking accounted for 1.21%. Passive smokers accounted for 62.44 and 82.17% of them were exposed to cooking oil smoke almost every day. Additionally, 11.06% of the participants had a history of alcohol consumption, and 1.02% reported current alcohol consumption. Moreover, 85.82% did not often participate in outdoor physical exercise, and 92.70% did not drink tea. More than 50% of the women had an insufficient intake of fresh vegetables and fruits with vegetable intake < 5 kg per week, and fruit intake < 2.5 kg per week. Approximately 70% of women did not meet the dietary guideline requirements set forth for Chinese residents (Table 3).

**TABLE 3:** Living conditions of the cervical cancer screening population.

Characteristics		N	n (%)
Smoking	No	5,390	90.42
	Currently smoking	499	8.37
	Smoked before	72	1.21
Second hand smoke	Yes	3,722	62.44
	No	2,239	37.56
Cooking fumes	Almost everyday	4,898	82.17
	Sometimes	951	15.95
	Almost not	112	1.88
Alcohol consumption	No	6,241	104.70
	Currently drinking alcohol	61	1.02
	Previously drank alcohol	659	11.06
Exercise	Yes	845	14.18
	No	5,116	85.82
Tea drinking	Yes	435	7.30
	No	5,526	92.70
Vegetable consumption	Never	212	3.56
	<5 Pounds/week	3,407	57.15
	≥5 Pounds/week	2,317	38.87
Fruit consumption	Never	209	3.51
	<2.5 Pounds/week	3,462	58.08
	≥2.5 Pounds/week	2,274	38.15
Livestock meat consumption	Never	258	4.33
	≤350 g/Week	4,578	76.80
	>350 g/Week	1,109	18.60
Coarse grain consumption	Never	617	10.35
	<1 Pound/week	4,731	79.37
	≥1 Pound/week	591	9.91

#### Health history

Of the participants, 60.69% had a good health status or self-rated their health status as good. An additional 9.21% had a history of hypertension, 3.62% of diabetes, 11.84% of hyperlipidemia, and 0.22% of mental health illness. Moreover, 20.33% of the participants experienced negative life events, 32.52% had depression or anxiety symptoms, and 35.31% had poor sleep quality (Table 4).

**TABLE 4:** Health-related emotional factors of the cervical cancer screening population.

Characteristics		N	n (%)
Self-assessed health status	Very good or good	3,618	60.69
	General	1,947	32.66
	Not good	396	6.64
Hypertension	Yes	549	9.21
	No	5,412	90.79
Diabetes	Yes	216	3.62
	No	5,745	96.38
Hyperlipidemia	Yes	706	11.84
	No	5,255	88.16
Diagnosed with a mental illness	Yes	13	0.22
	No	5,948	99.78
Experienced a negative life event	No	4,749	79.67
	1–2 Events	1,160	19.46
	≥3 Events	52	0.87
Mental depression	No	4,111	68.96
	Occasionally	1,461	24.51
	>1 Month	186	3.12
	>6 Months	203	3.41
Anxiety	No	4,022	67.47
	Occasionally	1,633	27.39
	>1 Month	191	3.20
	>6 Months	115	1.93
Sleep quality	Good	3,856	64.69
	Difficult to fall asleep	524	8.79
	Wake up early	535	8.98
	Sleep well	960	16.10
	Wake up at night	86	1.44
People offering support in difficult times	Husband	5,371	90.10
	Parents	3,255	54.60
	Children	3,954	66.33
	Siblings	3,300	55.36
	Friends	3,100	52.00
	Colleagues	1,203	20.18
	No support	68	1.14

#### Population screening willingness

A total of 22.38% of the participants believed they could easily develop cancer, while 31.74% had been previously screened for cancer. Cancer screening for 27.38% of the participants was paid for by the government. Moreover, 28.07% indicated that they would fully accept cancer screening. Those who did not accept cancer screening listed time concerns and no conscious symptoms.

In the case of abnormal inspection results, most women were willing to be re-inspected and felt they were able to cover the associated expenses. However, the acceptance rate for self-paid expenses was typically below 200 Yuan. Moreover, 92.35% of the participants indicated that they were willing to accept a follow-up visit in the case of abnormal results. The primary reason preventing participants from accepting subsequent appointments was the time requirement.

Additionally, 91.01% of the participants indicated that they were willing to try new, more effective screening technologies, with acceptable out-of-pocket expenses for these technologies concentrated below 200 Yuan. The participants who were unwilling to accept new, more effective screening technologies, primarily showed concern for the scientific validity and safety of the new methods (Table 5).

**TABLE 5:** Screening willingness of the cervical cancer screening population.

Characteristics		N	n (%)
Do you think you are prone to cancer?	Yes	1,334	22.38
	No	4,532	76.03
Have you ever been screened for cancer?	Yes	1,892	31.74
	No	3,987	66.88
Who bears the cost of cancer screening?	It is all borne by the government and not paid by individuals	1,632	27.38
	Some expenses shall be borne by individuals	199	3.34
	All expenses shall be borne by individuals	40	0.67
	No idea	42	0.70
To what extent do you accept cancer screening?	Totally acceptable	1,673	28.07
	Acceptable	218	3.66
	Difficulty in accepting	9	0.15
	Unacceptable	0	0.00
Reasons for not participating in cancer screening	Economic reasons	1,556	26.10
	Time reason	2,776	46.57
	The procedure is cumbersome and laborious	1,350	22.65
	Examination can cause pain	1,719	28.84
	I do not think there are any symptoms in my body. It’s unnecessary	1,787	29.98
	Physical condition does not allow	31	0.52
	Unaccompanied	52	0.87
If the examination result is abnormal, would you be willing to be checked again?	Yes	5,530	92.77
	No	380	6.37
Would you like to be checked again?	Yes	5,166	86.66
	No	276	4.63
What is an acceptable examination fee for you?	<100 Yuan	537	9.01
	100–199 Yuan	2,470	41.44
	200–299 Yuan	985	16.52
	≥300 Yuan	1,165	19.54
Are you willing to make a return visit?	Yes	5,505	92.35
	No	373	6.26
Reasons for not willing to make a return visit/recheck.	Economic reasons	619	10.38
	Time reason	887	14.88
	The inspection is cumbersome and laborious	582	9.76
	Examination can cause pain	208	3.49
	I do not think there are any symptoms in my body. It is unnecessary	149	2.50
	Physical condition does not allow	17	0.29
	Unaccompanied	7	0.12
Are you willing to accept new technology?	Yes	5,425	91.01
	No	478	8.02
How much are you willing to pay for the new technology?	<100 Yuan	671	11.26
	100–199 Yuan	2,628	44.09
	200–299 Yuan	950	15.94
	≥300 Yuan	1,121	18.81
Reasons for reluctance to accept new technology screening.	Question the scientific validity and safety of the new method	1,145	19.21
	Unclear interpretation and utilization of screening results	697	11.69
	High cost	512	8.59
	The old method is reliable, there is no need to use the new method	112	1.88
	Concerned about the pain of new screening methods	4	0.07

#### Gynecological examination

All participants (5,961) completed gynecological examinations for cervical cancer screenings (Table 6). The detection rates of leukoplakia, ulcers, condyloma, and tumors were 0.34, 0.02, 0.03, and 0.05%, respectively. The detection rates of congestion, condyloma, and tumors in vaginal examinations were 1.16, 0.02, and 0.02%, respectively. The rates of tofu-like, purulent, foam, blood, and peculiar smell were 0.12, 1.69, 0.82, 0.10, and 0.55%, respectively, whereas the rates of cervical hypertrophy, ulcers, erosion, atrophy, polyps, and cysts were 1.93, 0.12, 5.54, 10.97, 2.89, and 9.56%, respectively.

**TABLE 6:** Gynecological examination of the cervical cancer screening population.

Characteristics		N	n (%)
Vulva	Normal	5,810	97.47
	Vitiligo	20	0.34
	Ulcer	1	0.02
	Condyloma	2	0.03
	Tumor	3	0.05
	Other	32	0.54
Vaginal	Normal	5,773	96.85
	Congestion	69	1.16
	Condyloma	1	0.02
	Tumor	1	0.02
	Other	24	0.40
Secretion	Normal	5,475	91.85
	Tofu-like	7	0.12
	Purulent	101	1.69
	Foam	49	0.82
	Bloody	6	0.10
	Peculiar smell	33	0.55
	Excessive	157	2.63
	Other	40	0.67
Cervix	Normal	3,646	61.16
	Hypertrophy	115	1.93
	Congestion	48	0.81
	Touch blood	24	0.40
	Ulcer	7	0.12
	Erosion	390	6.54
	Shrink	654	10.97
	Polyp	172	2.89
	Cervical cyst	570	9.56
	IUD tail wire	24	0.40
	Other	217	3.64
Total		5,961	100.00

### Human papillomavirus and thinprep cytology test distribution

**FIGURE 1:**
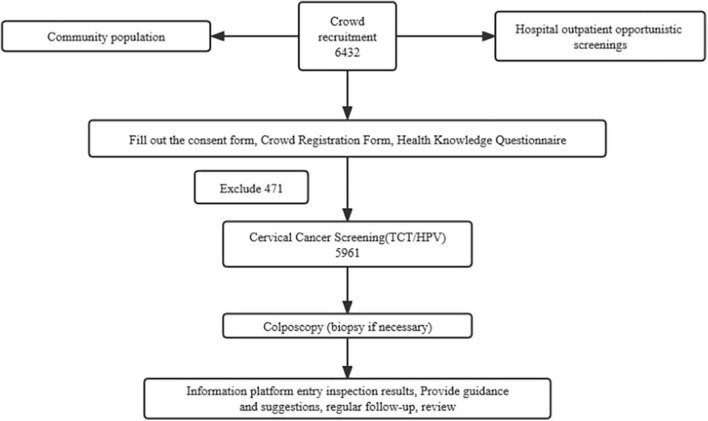
The CONSORT flow of study.

Of the 6,432 participants, 471 were excluded from the survey results. The remaining 5,961 participants were screened for cervical cancer and included in the statistical analysis (Table 7). The average age of the remaining participants was 51.51 ± 6.61 years, with a median age of 51 years (Figure 1). Cytological examinations revealed 562 cases that were TCT positive; 9.43% (562/5961) were ASCUS +, of which 6.71% had ASCUS (not statistically significant), an additional 0.50% had ASC-H, 1.63% had LSIL, 0.27% had HSIL, and 0.22% had AGC-NOS. Additionally, AGC-N was reported in 0.10% of the participants, while SCC, cervical carcinoma *in situ*, and adenocarcinoma were not detected. Moreover, TCT-/HPV + accounted for 7.68% of the cases, TCT+/HPV- for 4.71%, and TCT + /HPV + for 4.71%.

**TABLE 7:** Thinprep cytology test (TCT) and HPV cervical cancer screening results.

TBS classification diagnostic criteria	N	HPV (+)	HPV (–)	n (%)
NILM	5,399	458	4,941	7.68
ASC-UC	400	154	246	2.58
ASC-H	30	28	2	0.47
LSIL	97	79	18	1.33
HSIL	16	15	1	0.25
SCC	0	0	0	0.00
AGC-NOS	13	3	10	0.05
AGC-N	6	2	4	0.03
AIS	0	0	0	–
Adenocarcinoma	0	0	0	–
Total	5,961	739	5,222	–

TBS, the Bethesda system; NILM, negative for intraepithelial lesion or malignancy; ASCUC, atypical squamous cells of undetermined significance; ASC-H, atypical squamous cells cannot exclude high-grade squamous intraepithelial lesion; LSIL, low-grade squamous intraepithelial lesion; HSIL, high-grade squamous intraepithelial lesion; SCC, squamous cell carcinoma; AGC-NOS, atypical glandular epithelial cells-not otherwise specified; AGC-N, atypical glandular epithelial cells-not prone to cancer; AIS, adenocarcinoma in situ.

Of the 5,961 participants, 739 (12.40%) tested positive for HPV (Table 8), of which 106 were HPV-16 positive (14.34, 1.78% positive rate), 46 were HPV-18/45 positive (6.22, 0.77% positive rate), and 589 were positive for other high-risk types of HPV genotypes (79.70; 9.88% positive rate). The positive rate of HPV-16 and HPV-18/45 in the total screening population was 2.52%. Two of the participants tested positive for both type 16 and type 18/45.

**TABLE 8:** Human papillomavirus (HPV) screening results in cervical cancer.

HPV		N	n (%)
Detection	(+)	739	12.40
Condition	(–)	5,222	87.60
Typing	16 type	106	1.78
	18/45 type	46	0.77
	Other	589	9.88
Total		5,961	100.00

### Age specificity of thinprep cytology test positive and high-risk-human papillomavirus infection

The 5,961 participants that were enrolled for TCT and HR-HPV screening from December 2018 to December 2021 were divided into six age groups, and the positive detection rates were determined for each. It was found that 35–40, 41–45, 46–50, 51–55, 56–60, and 61–65 year old participants had TCT positive detection rates of 9.92, 10.51, 10.94, 9.36, 7.71, and 6.95%, respectively (Table 9). The prevalence of HR-HPV infection reached two peaks at the ages of 56–60 and 61–65, with infection rates of 14.71 and 15.51%, respectively (Table 10).

**TABLE 9:** Age specificity of TCT positivity.

TBS classification diagnostic criteria	35–40 (year)	41–45 (year)	46–50 (year)	51–55 (year)	56–60 (year)	61–65 (year)
NILM	236	842	1,408	1,308	1,029	576
ASC-UC	16	72	121	99	65	27
ASC-H	3	3	7	7	6	4
LSIL	6	17	31	23	13	7
HSIL	0	5	3	3	1	4
SCC	0	0	0	0	0	0
AGC-NOS	1	2	8	1	1	0
AGC-N	0	0	3	2	0	1
AIS	0	0	0	0	0	0
Adenocarcinoma	0	0	0	0	0	0
Total	262	942	1,581	1,443	1,115	619
ASCUS +	26	99	173	135	86	43
Positive n (%)	9.92%	10.51%	10.94%	9.36%	7.71%	6.95%

**TABLE 10:** Age specificity of human papillomavirus (HPV) infection.

Year	N	HPV(+)	HPV(–)	n (%)	χ^2^	*P*
35–40	262	29	233	11.07	15.9	0.0071
41–45	941	101	840	10.73		
46–50	1,581	184	1,397	11.64		
51–55	1,443	165	1,278	11.43		
56–60	1,115	164	951	14.71		
61–65	619	96	523	15.51		

### Single-factor analysis of risk factors for human papillomavirus infection

A total of 28 potential risk factors may be related to HR- HPV infection. Univariate logistic analysis showed that HPV infection was correlated with different age groups, educational level, age at first sexual encounter, number of sexual partners, contraceptive methods, TCT positivity, nationality, and population (*p* < 0.05; Table 11).

**TABLE 11:** Comparison of information and living habits of women with a positive HPV detection rate.

Characteristics		N	(+)	(–)	n (%)	χ^2^	*P*
Age (years)	35–45	1,203	130	1,073	10.81	15.61	0.0004
	46–55	3,024	349	2,675	11.54		
	56–65	1,734	260	1,474	14.99		
Family history of cancer	Yes	987	139	848	14.08	3.095	0.0785
	No	4,974	600	4,374	12.06		
Level of education	High school and below	3,999	544	3,455	13.60	16.22	0.0001
	College degree or above	1,961	195	1,766	9.94		
Age at initial sexual experience	≤19 Years	179	31	148	17.32	4.04	0.0444
	>19 Years	5,723	703	5,020	12.28		
Number of sexual partners	Multiple (≥2)	532	85	447	15.98	6.704	0.0096
	1	5,375	650	4,725	12.09		
Number of abortions	>1	1,949	249	1,700	12.78	0.9141	0.3390
	1	2,279	314	1,965	13.78		
Number of deliveries	>1	544	78	466	14.34	1.518	0.2179
	≤1	5,124	640	4,484	12.49		
Number of marriages	>1	320	45	275	14.06	0.8146	0.3668
	≤1	5,587	690	4,897	12.35		
Contraceptive methods used	Others	4,508	582	3,926	12.91	7.283	0.0070
	Condom	727	68	659	9.35		
Sexual partner has long foreskin	Yes	323	40	283	12.38	0.001146	0.9728
	No	5,567	693	4,874	12.45		
Bleeding during intercourse	Yes	385	53	332	13.77	0.6647	0.4149
	No	5,499	679	4,820	12.35		
Leucorrhea abnormality	Yes	679	73	606	10.75	1.988	0.1585
	No	5,146	651	4,495	12.65		
Vaccination	No	759	535	224	70.49	–	> 0.999
	Yes	3	2	1	66.67		
Extramarital sex	Yes	63	10	53	15.87	0.6793	0.4098
	No	5,819	723	5,096	12.42		
Ethnicity	Han	5,749	699	5,050	12.16	8.661	0.033
	Others	211	40	171	18.96		
Total household income	≤50,000 Yuan	3,188	429	2,759	13.46	4.147	0.0417
	>50,000 Yuan	2,120	245	1,875	11.56		
Breastfed	No	1,005	120	885	11.94	0.2324	0.6297
	Yes	4,956	619	4,337	12.49		
Smoking history	Yes	571	63	508	11.03	1.082	0.2983
	No	5,390	676	4,714	12.54		
Alcohol consumption	Yes	720	82	638	11.39	0.7667	0.3812
	No	5,241	657	4,584	12.54		
Physical exercise	Yes	845	118	727	13.96	2.227	0.1356
	No	5,116	621	4,495	12.14		
Tea drinking	No	5,526	692	4,384	12.52	2.76	0.0966
	Yes	435	47	388	10.80		
Fresh vegetable consumption	No	3,619	451	3,168	12.46	0.03397	0.8538
	Yes	2,317	285	2,032	12.30		
Fresh fruit consumption	No	3,671	449	3,222	12.23	0.1968	0.6573
	Yes	2,274	287	1,987	12.62		
Meat consumption	No	4,836	595	4,241	12.30	0.3845	0.5352
	Yes	1,109	144	965	12.98		
Coarse grain consumption	No	5,348	659	4,689	12.32	0.6942	0.4047
	Yes	592	80	512	13.51		
Are you in good health?	No	396	60	336	15.15	2.963	0.0852
	Yes	5,565	679	4,886	12.20		
TCT	ASCUS and above	562	281	281	50.00	807.9	< 0.0001
	NILM	5,399	458	4,941	8.48		
Population area	Urban population	3,961	463	3,498	11.69	5.453	0.0195
	Rural population	2,000	276	1,724	13.80		

TCT, Thinprep cytology test; ASCUS, atypical squamous cells of undetermined significance; NILM, negative for intraepithelial lesion or malignancy.

### Logistic regression analysis of risk factors of human papillomavirus infection

The results of the multivariate unconditional logistic regression analysis showed that high school education or lower [odds ratio (OR) = 1.426 (1.199–1.696), *p* < 0.001], age at first sexual encounter ≤ 19 years [OR = 1.496 (1.008–2.220), *p* = 0.046], number of sexual partners > 1 [OR = 1.382 (1.081–1.768), *p* = 0.010], assigned TBS classification of ASCUS or above [OR = 10.788 (8.912–13.060), *p* < 0.001], non-condom-based contraception [OR = 1.437 (1.103–1.871), *p* = 0.007], other nationalities except Han [OR = 1.690 (1.187–2.406), *p* = 0.004], and rural residence [OR = 1.210 (1.031–1.419), *p* = 0.020] were risk factors for HPV infection (Table 12). Compared with women aged 56–65, the infection rates in women aged 35–45 [OR = 0.687 (0.549–0.860), *p* = 0.001] and 46–55 [OR = 0.740 (0.622–0.8709), *p* = 0.001] were significantly reduced.

**TABLE 12:** Multivariate analysis of risk factors affecting human papillomavirus (HPV) infection.

Characteristics	SE	*P*	OR (95%)
35–45 year	0.115	0.001	0.687 (0.549∼0.860)
46–55 year	0.088	0.001	0.740 (0.622∼0.879)
High school and below	0.088	<0.001	1.426 (1.199∼1.696)
Initial age of sexual life ≤ 19 years	0.202	0.046	1.496 (1.008∼2.220)
Number of sexual partners > 1	0.126	0.010	1.382 (1.081∼1.768)
ASCUS and above	0.097	<0.001	10.788 (8.912∼13.060)
Contraceptive methods other than condoms	0.135	0.007	1.437 (1.103∼1.871)
Nationalities other than Han	0.180	0.004	1.690 (1.187∼2.406)
Rural population	0.082	0.020	1.210 (1.031∼1.419)

OR, odds ratio; ASCUS, atypical squamous cells of undetermined significance; HSIL, high-grade squamous intraepithelial lesion.

## Discussion

Currently, cervical cancer is the second most common female malignant tumor after breast cancer. The incidence rate of cervical cancer has increased significantly and has become increasingly prevalent in younger women. As such, establishing effective preventative and therapeutic strategies for cervical cancer has become increasing important. Various studies (Sui et al., 2018) have reported that HPV infection represents the primary biological cause of cervical precancerous lesions and cervical cancer. The pathological process from HPV infection, to cervical precancerous lesion development, to carcinoma, to invasive carcinoma *in vivo*, typically occurs over a span of approximately 10 years. Hence, interrupting the early stages of HPV infection, or early stages of cervical lesion formation may effectively prevent, or delay, the occurrence and development of cervical lesions and cervical cancer.

A recent study (Schettino et al., 2008) found that the positive rate of HPV infection was closely related to geographical region, population, age, and living habits, among other factors. In particular, HPV infection was found to have a strong regional characteristic, as the positivity rate and distribution of HPV type varies in different countries or regions around the world. That is, regions with relatively developed economies exhibit lower positivity rates. For instance, a study in South Korea, comprising 18,170 women had a HR-HPV positive rate of 12.5% (Ouh et al., 2018). Similarly, a study in Turkey showed that the incidence rate of HR-HPV infection was ∼9.17% (Taskin et al., 2022). Meanwhile, in countries with relatively underdeveloped economies, such as sub-Sahara Africa and Bangladesh, the HR-HPV infection rate was 34% (Seyoum et al., 2022) and ∼41.86% (Sharmin et al., 2021), respectively. Moreover, although the total positivity rate of HPV infection in China is ∼21.69%, it varies between economically developed and underdeveloped areas. For instance, a study in Shanghai included 6,619 women, of whom the HR-HPV infection rate was 9.5% (Wang et al., 2021), whereas another study in Jiangsu included 36,500 women and reported a 28.95% HR-HPV infection rate (Niu et al., 2022). Still further, a study in Guangxi from 2016 to 2021 included 41,140 women, for whom the overall HR-HPV infection rate was 18.10% (Wei et al., 2022). In the current study, 5,961 cases of HPV were detected in urban and rural women in Shenyang, of which 739 were HPV positive, accounting for 12.40% of the women (739/5961). Significant regional differences were observed in HPV infection rates. These epidemiological studies on HR-HPV distribution and prevalence in different geographical regions can aid the development of strategies to prevent HPV-related cancer, especially with regard to vaccines (Smith et al., 2008; Wang et al., 2019). Early diagnosis, the use of preventive HPV vaccines, and screening tests are critical for the prevention of precancerous lesions and cervical cancer (Alotaibi et al., 2020).

Age differences were also observed with regard to HPV infection rate, in the current study. Previous studies have reported that HPV infection is more common in elderly women (Hariri et al., 2011). Here, we examined the prevalence of HPV in six age groups and found the highest prevalence in women aged 61–65, at a rate of 15.51%. Similarly, women aged 55–60 showed a positive infection rate of 14.7%. The TCT positive rate was lower in the 51–55 and 56–65 age groups; however, their HR-HPV positivity rates were higher than those of the other age groups. These results may have been missed TCT diagnoses, which can result from the inward movement of the cervical squamous column junction in perimenopausal women. The close relationship between HPV infection rate and age may be due to the development of clearance over time, with changes in sexual activity and immunity acquired from previous infections (Arbyn et al., 2011). The increased prevalence of HPV in the elderly can further be explained by the reactivation of potential infection (Giuliano et al., 2019). Therefore, a cervical HPV screening program is particularly important for perimenopausal women (Shi et al., 2017; Li et al., 2020). In women of all ages, the positive rate of HPV infection showed a “U” shaped change. The first peak of infection typically occurred during the early stages of their sexual life, which was likely related to factors that affect the cervical self-clearance ability. These factors include the age at first sexual encounter, immature cervical development, failure to practice safe sex, frequent sex, multiple sexual partners, and personal health problems. Meanwhile, the second peak occurred primarily during perimenopause, which may be related to a change in sex hormone levels in aging women, a decline in immune function, a decrease in cervical resistance, an increase in virus susceptibility, or the reactivation of latent viral infection. Therefore, it is important to continue promoting the benefits of safe sex, while also implementing public health education resources related to health issues, individual immunity, healthy habits, and the importance of regular health checks. Moreover, the use of endocrine therapy by perimenopausal women may reduce the incidence of HPV infection. Hence, more attention should be dedicated to screening for HPV infection in elderly women.

Previous studies have reported that first sexual encounters occurring before the age of 19 years represents an independent risk factor for HR-HPV infection. The cervical epithelial repair function and autoimmune function of women under 19 years of age are not yet fully mature, rendering them susceptible to HR-HPV (Liu et al., 2015). Moreover, puberty hormones can promote HPV infection, with the hormone concentration in women younger than 19 years being relatively high. Sexual intercourse serves as the primary route of HPV transmission, while having multiple sexual partners increases the risk of HPV infection in women. Other studies have shown an association between the number of sexual partners and the risk of cervical cancer (Kitamura et al., 2021), a finding that was also demonstrated by the international cooperation, comprising 21 epidemiological studies (International Collaboration of Epidemiological Studies of Cervical Cancer, 2009). Here, we also found an association between the number of sexual partners and the risk of HVP infection. Although studies have shown that an active sexual life is closely related to the occurrence and development of cervical cancer, condoms can block the destruction of cervical mucosa by pathogens, inhibit immune function, reduce the probability of infection, reduce the cervical mucosa stimulation by semen, and help reduce HPV transmission (Hariri and Warner, 2013).

Consistent with other studies (Niu et al., 2022), the current study shows that women with a high school education or lower, have a higher risk of HPV infection than women with a higher education level. Additionally, socioeconomic status is closely related to the incidence of cervical cancer, particularly in developing countries. A higher education level may improve an individual’s awareness of beneficial health practices, thus reducing their risk of disease (Sharmin et al., 2021). Hence, education pertaining to HPV and the HPV vaccine should be strengthened to improve self-protection awareness.

This study also found that living in rural areas represents a high-risk factor for HPV infection, which may be due to a lack of cervical cancer screening services, poverty in rural areas, and poor education. Indeed, Vhuromu et al. (2018) reported that the utilization rate of cervical cancer screening in rural areas is low. Hence, new approaches to improve health systems and cervical cancer screening services in rural areas may improve these disparities, thereby reducing cervical cancer cases in rural areas.

An international study conducted by IARC in 2011 showed that compared to those who never smoked, the risk of cervical cancer for current smokers was OR = 1.94 (95% confidence interval: 1.26–2.98) (Louie et al., 2011); however, no significant difference was observed between women that smoked and those that did not.

Currently, HPV vaccine research (preventive and therapeutic) has significantly increased. A study in the United States found that HPV preventive vaccines (bivalent, tetravalent, and non-valent vaccines) are safe and have preventive and protective effects (Blake and Middleman, 2017). However, given that different countries and regions have different HPV infection conditions, the vaccine benefits may vary based on geographical location. As such, China should focus on developing an HPV-specific preventive vaccine suitable for Chinese nationals, which takes into consideration the dual factors of regional typing and age stratification during vaccination.

## Conclusion

The occurrence and development of cervical cancer is a multi-factor, complex, and progressive biological process. Currently, the relationship between HPV infection, cervical cancer, and precancerous lesions is clear. Through cervical screening of female HPV infection rate, this study identified that a high school education or lower, age at first sexual encounter ≤ 19 years, number of sexual partners > 1, TBS classification of ASCUS or above, non-condom-based contraception, other nationalities except Han, and rural population serve as risk factors for HPV infection. It is, therefore, necessary to establish and improve the cervical cancer screening system according to the relevant factors affecting the incidence of cervical cancer, screen out and follow-up with high-risk groups, strengthen public education on prevention and treatment of cervical cancer, and improve awareness of cancer prevention among the general population. Moreover, targeted improvement measures for women in different regions are fundamental to reducing the positivity rate of HPV infection. Improving the living environment, improving education level, expanding health education, developing beneficial health habits, and actively preventing and treating HPV infection are the basic and effective ways to reduce the positive rate of HPV infection. Only by providing a large amount of epidemiological data can we carry out effective vaccine research and development while also optimizing the cost-effectiveness of vaccinating the Chinese population. Collectively, the results of this study provide epidemiological data for the development of an HPV vaccine, to effectively reduce the incidence of cervical cancer in Shenyang.

## Data availability statement

The raw data supporting the conclusions of this article will be made available by the authors, without undue reservation.

## Ethics statement

This study was approved by the Ethics Committee of the Liaoning Cancer Hospital (Ethics batch number: 20180106), and informed consent was obtained from all participants included in this study. The patients/participants provided their written informed consent to participate in this study. Written informed consent was obtained from the individual(s) for the publication of any potentially identifiable images or data included in this article.

## Author contributions

HP and CW designed and supervised the project. DY, JZ, and XC collected the clinical data samples. DY collected and processed the data, performed the data analysis, and drafted the manuscript. JM translated and edited the manuscript. All authors reviewed, discussed and edited version of the manuscript, and agreed with the final manuscript.
